# Supplementary material for: Acidic C-terminal domains autoregulate the RNA chaperone Hfq
Source: eLife. 2017 Aug 9;6:e27049. doi: 10.7554/eLife.27049 (PMC5606850; doi:10.7554/eLife.27049)
Supplement: Supplementary file 1. — Step-by-step description of FloppyTail simulations, with a fully worked example on E. coli Hfq. [file elife-27049-supp1.pdf]

Hfq Rosetta FloppyTail Supplemental Protocol

Materials and Equipment

- Mac OS X or Linux
- Python 2.7
- Rosetta 2017.12 (https://www.rosettacommons.org/downloads/academic/2017/wk12)
- PyRosetta4 (http://www.pyrosetta.org/dow)

Procedure

Generating the Extended Tail Structure

1. Acquire the *E. Coli* Hfq, or other desired, crystal structure. A little post-processing may be necessary. In this case, we need to unzip the file. In other cases, it may be necessary to generate the hexameric structure from the symmetry operations reported in the PDB.

```
$ wget http://www.rcsb.org/pdb/files/1HK9.pdb.gz
```

2. Clean the structure of non-ATOM records:

```
$ grep ATOM 1hk9.pdb > 1hk9.clean.pdb
```

3. Use the `extend_terminus.py` script to append/prepend the termini sequences, one monomer at a time. This will set ϕ/ψ values to -135°/135° for the appended/prepended residues. For example, to prepend "MAKGQ" to the N-terminus of chain A, use the following command:

```
$ extend_terminus.py -c A -o 1hk9.Ap.pdb -p 1hk9.clean.pdb MAKGQ
```

The arguments break down as follows:

   a. The `-c` flag with the `A` argument specifies the chain.
   b. The `-p` flag indicates prepend mode, `-a` should be used to append.
   c. The `-o` flag with the `1hk9.Ap.pdb` argument specifies the output file.
   d. The positional argument `1hk9.clean.pdb` specifies the input file.
   e. Finally, the positional argument `MAKGQS` defines the sequence to prepend, with a single residue overlap.

To prepend all six N-termini, repeat the above command for each chain, using the previous output as input. For example, to prepend to chain B, using the structure produced by prepending to chain A, we would run:

```
$ extend_terminus.py -c B -o 1hk9.Bp.pdb -p 1hk9.Ap.pdb MAKGQS
```

*note the sequence may change if subunits are not identical!
An alternative approach to independently extend all termini would be to extend the termini of a single subunit and then generate C6 symmetric copies of the monomer to produce the hexamer (this approach ensures identical starting conformations for all subunits). Note that step 4 should be carried out prior to creating a symmetric hexamer from a single monomer, as step 4 fixes the backbone dihedral angles in tail residues that were resolved in the X-ray crystal structure.

4. To generate models with identical starting positions, we use the `convert_to_beta.py` script. The script converts all backbone dihedral angles to the ideal values (ϕ = -135, ψ = 135) for an extended conformation, given a direction (either toward the c-terminus, `-c`, or the n-terminus, `-n`) and a starting residue (`-s`). Additionally, the `--publication-specific` flag can be used to apply dihedral angles from the *E. coli* Hfq structure (residues 65–70) instead of ideal angles. By default, the script iterates over all chains.

```
$ convert_to_beta.py -i 1hk9.pdb -s 65 -c -o 1hk9.extend.pdb --
publication-specific
```

5. Once the structure containing all disordered regions has been generated, it needs to be "relaxed" in Rosetta's all-atom energy function. Relaxing alleviates defects and running simulations on unrelaxed input structures is bad practice as it makes interpreting results more difficult. Here, relax with restraints is used as we don't want to move the termini at this stage:

```
path/to/Rosetta/main/source/bin/relax.macosclangrelease \
      -s 1hk9.extend.pdb \
      -relax:constrain_relax_to_start_coords \
      -relax:ramp_constraints false \
      -ex1 \
      -ex2 \
      -use_input_sc  \
      -flip_HNQ \
      -no_optH false \
      -relax:min_type lbfgs_armijo_nonmonotone \
      -nstruct 1
```

The necessary flags and arguments here are `-s input.pdb`, `-relax:constrain_relax_to_start_coords`, and `-relax:ramp_constraints false`. These specify the input structure, set harmonic restraints to the starting backbone atomic positions, and turn off restraint ramping (respectively). The other flags arguments are good practice. The flags, `-ex1` and `-ex2`, increase sampling, by one standard deviation, of the first and second side-chain dihedral angles, while `-use_input_sc` includes the crystallographic side-chain conformation in the rotamer set sampled during relax. The flag, `-flip_HNQ`, tests alternate configurations for specific atoms (which cannot be distinguished in the electron density) in the side-chains histidine, asparagine, and glutamine. The flag and argument, `-no_optH false`, turn off hydrogen optimization during relax. Finally, the flag, `-relax:min_type`, specifies the minimization algorithm to be used during relax. Additionally, `-nstruct N` can be used to generate a number, N, of structures. Due to restraints, there is little structural variation, so one will suffice.

Running Rosetta FloppyTail

The FloppyTail algorithm is fully described in Kleiger *et al.*[1] and demonstrated to be useful for studying disordered regions in both Crawley *et al*[2]. and Zhang *et al.*[3]

[1] http://www.ncbi.nlm.nih.gov/pubmed/19945379
[2] http://www.ncbi.nlm.nih.gov/pubmed/21071445
[3] http://www.ncbi.nlm.nih.gov/pubmed/23395180

Briefly, FloppyTail is a Monte Carlo algorithm that samples conformations of a disordered region in two phases. The first is a low resolution phase where the backbone atoms are fully represented, but side-chains are “summarized” by a single pseudo-atom centroid centered at the Cβ atom position. In this phase, large changes in the backbone dihedral angles, ϕ/ψ, are attempted (known as small/shear moves) and accepted in accordance with the Metropolis criterion. Occasionally (3% of the time), the energy is minimized using a gradient-based minimization. In the second phase, side-chains centroids are restored to their fully atomic representations. Then, side-chain dihedrals are sampled as well as backbone dihedrals. Furthermore, side-chain repacking (describe better) precludes every other gradient-based minimization move.

To set the myriad options of FloppyTail, we use a “flag file” (sample for monomer):

```
# input
-s 1hk9.relax.pdb

# define flexible region via movemap file
-movemap movemap.txt

# shear moves are not productive initially so no shear
# for the first 1/3 of the simulation
-FloppyTail::shear_on 0.333

# root the fold tree at the center of mass, so
# we can “flop” both termini simultaneously
-FloppyTail::COM_root

# do not change AA identities during packing
-packing::repack_only

# monte carlo and sampling options
-FloppyTail::perturb_temp 0.8
-FloppyTail::perturb_cycles 100000 # ~500 moves per residue
-FloppyTail::refine_temp 0.8
-FloppyTail::refine_cycles 1000
-FloppyTail::refine_repack_cycles 10

# current best practices for minimization/scoring
-run::min_type lbfgs_armijo_nonmonotone
-score::weights talaris2014

# recommended number of structures to model
-nstruct 30000

# output
-out:path:pdb decoys/
-out:pdb_gz
```

The movemap file defines degrees of freedom for specified regions. Below, we give an example for *E. coli* Hfq, in Rosetta numbering (residues are continuously number from 1, as they appear in the PDB file).

```
RESIDUE * CHI # repack only, default for all
JUMP * NO # do not move subunits relative to one another
# chain A
RESIDUE 1 5 BBCHI
RESIDUE 65 102 BBCHI
# chain B
RESIDUE 103 107 BBCHI
RESIDUE 167 204 BBCHI
# chain C
RESIDUE 205 209 BBCHI
RESIDUE 269 306 BBCHI
# chain D
RESIDUE 307 311 BBCHI
RESIDUE 371 408 BBCHI
# chain E
RESIDUE 409 413 BBCHI
RESIDUE 473 510 BBCHI
# chain F
RESIDUE 511 515 BBCHI
RESIDUE 575 612 BBCHI
```

The simulation is then carried out on a cluster (either the Maryland Advanced Research Computing Center, MARCC, or Jazz, our in-house cluster). Using 720 cores, in parallel, the monomer simulation takes 48–64 hours. Below is a sample submission script for the slurm queue management system used by MARCC.

```
#!/bin/bash -l

#SBATCH --job-name=HFQ_FT
#SBATCH --partition=parallel
#SBATCH --time=80:0:0
#SBATCH --nodes=30
#SBATCH --ntasks-per-node=24
#SBATCH --mem=120GB
#SBATCH --output /dev/null # do not want verbose FT output
#SBATCH --error outerr/%j.err  # Name of stdout output file (%j
expands to jobId), remember to make outerr directory
#SBATCH --mail-user=jeliazkov@jhu.edu
#SBATCH --mail-type=END

# loading and unloading modules, for MPI
module unload openmpi/intel/1.8.4 gcc python
module load intel-mpi git
module load anaconda-python

# job description
ROSETTABIN=/home-2/jjeliaz1@jhu.edu/Rosetta/main/source/bin
ROSETTAEXE=FloppyTail
COMPILER=mpi.linuxgccrelease
EXE=$ROSETTABIN/$ROSETTAEXE.$COMPILER
echo Starting MPI job running $EXE

# running with a date and time stamp
date
time mpirun $EXE @floppy_tail.flags
date
#
```

Analysis

Tail–Core Interaction Calculations

The script `identify_interactions.py` can be used to identify residue–residue interactions between two sets of residues over a set of models, or decoys.

```
identify_interactions.py --pdb_numbering --n_procs 14 --set1 66-82 -
-set2 1-65 --manual_suffix .pdb.gz decoys
```
